# Blood biomarker for Parkinson disease: peptoids

**DOI:** 10.1038/npjparkd.2016.12

**Published:** 2016-06-23

**Authors:** Umar Yazdani, Sayed Zaman, Linda S Hynan, L Steven Brown, Richard B Dewey, David Karp, Dwight C German

**Affiliations:** 1 Department of Psychiatry, University of Texas Southwestern Medical Center, Dallas, TX, USA; 2 Department of Clinical Sciences, University of Texas Southwestern Medical Center, Dallas, TX, USA; 3 Department of Neurology & Neurotherapeutics, University of Texas Southwestern Medical Center, Dallas, TX, USA; 4 Department of Internal Medicine, University of Texas Southwestern Medical Center, Dallas, TX, USA

## Abstract

Parkinson disease (PD) is the second most common neurodegenerative disease. Because dopaminergic neuronal loss begins years before motor symptoms appear, a biomarker for the early identification of the disease is critical for the study of putative neuroprotective therapies. Brain imaging of the nigrostriatal dopamine system has been used as a biomarker for early disease along with cerebrospinal fluid analysis of α-synuclein, but a less costly and relatively non-invasive biomarker would be optimal. We sought to identify an antibody biomarker in the blood of PD patients using a combinatorial peptoid library approach. We examined serum samples from 75 PD patients, 25 *de novo* PD patients, and 104 normal control subjects in the NINDS Parkinson’s Disease Biomarker Program. We identified a peptoid, PD2, which binds significantly higher levels of IgG3 antibody in PD versus control subjects (*P*<0.0001) and is 68% accurate in identifying PD. The PD2 peptoid is 84% accurate in identifying *de novo* PD. Also, IgG3 levels are significantly higher in PD versus control serum (*P*<0.001). Finally, PD2 levels are positively correlated with the United Parkinson’s Disease Rating Scale score (*r*=0.457, *P*<0001), a marker of disease severity. The PD2 peptoid may be useful for the early-stage identification of PD, and serve as an indicator of disease severity. Additional studies are needed to validate this PD biomarker.

## Introduction

Parkinson disease (PD) is the second most common neurodegenerative disease affecting an estimated 640,000 people age 65 years and over (1.6% of elders) in the United States.^1^ The incremental annual cost of PD was reported to be $10,349 per patient which, when combined with indirect costs owing to productivity losses and uncompensated family caregiver burden, resulted in a cost of $23 billion annually in the U.S. in 2005.^2^ Considering the 15% growth in the elderly population of the U.S. during the last decade, these costs can be expected to rise markedly in the years ahead as the population ages.

Patients with PD exhibit neurodegeneration in select groups of neurons and neuroinflammation, which is characterized by activated microglia and infiltrating T cells. As T cells activate B cells, which make antibodies, it is not surprising that there are PD-related antibodies in the serum of PD patients.^3–5^ The immune system may well have an important role in the progression of the disease, and immunotherapy may offer an approach to slow or stop disease progression.^6–8^ If we could ‘read’ immune responses in such a way that they could be linked to specific disease states, then a diagnostic tool of extraordinary utility would result. Furthermore, for disease states that are exacerbated by an immune response, if one could rapidly identify the offending antibodies and/or T-cells, and identify neutralizing molecules specific for them, a revolution in the treatment of these diseases would result.

Neuropathologically, PD is an inexorably progressive disorder of unknown cause affecting multiple neurotransmitter systems. In addition to the motor symptoms of the disease, non-motor features of the disease include autonomic failure, urinary incontinence, hallucinations, and dementia.^9^ Studies show that when PD first presents clinically, patients have lost ~60% of their striatal dopaminergic nerve terminals and ~30% of their nigral neurons.^10^ Because the disease is neuropathologically advanced at the time of diagnosis, discovery of a biomarker for the early identification of the disease is important for testing of disease modifying therapies. Brain imaging of the nigrostriatal dopamine system with radio-labelled cocaine analogs has demonstrated loss of dopaminergic terminals in the pre-motor phase of PD,^11^ but these radionuclide imaging results are influenced by dopaminergic treatments and are thus not suitable for tracking disease progression in treated PD patients.^12^ Identification of a serum biomarker for early diagnosis and monitoring of disease progression is a critical unmet need in the field.

Although a number of treatments have been developed that improve the ‘dopaminergic deficit’, no treatment has been demonstrated to slow neuronal degeneration. In human PD research, dopaminergic neurons in the substantia nigra cannot be counted in the live patient, so it is not possible to prove neuroprotection following a drug treatment. Thus, the demonstration of ‘disease-modification’ depends on measuring clinical features of disease progression as a surrogate. As reviewed in ref. 13, 15 clinical trials aimed at the goal of achieving disease modification in PD have been published, and although some were interpreted by the authors as being consistent with a neuroprotective effect, methodological limitations prevented the drawing of firm conclusions. Also, many putative disease-modifying therapies for PD also exert a symptomatic effect, which impacts clinical end points. Because of the inherent difficulties of using clinical outcome measures to assess disease modification, the identification of biomarkers for PD is of paramount importance. The ideal PD biomarker would be one that is abnormal during the presymptomatic phase of the illness, varies proportionally with disease severity, and is unaffected by drugs or other interventions used to treat PD. The data presented here describe a blood biomarker identified as part of the NINDS Parkinson’s Disease Biomarker Program (PDBP).^14^

## Results

The subjects used in the present study are described in Table 1. The PD group was composed of 75 subjects with a mean age of 69±5 years. The *de novo* group was composed of 25 subjects, mean of 62±10 years of age. The 104 normal control subjects were provided locally (*n*=21) and at another PDBP site (*n*=83), and they were age and gender matched with the PD group (mean age=69±5 years). The peptoid levels for the control subjects did not differ between the two sites (mean±s.d. for the 21 controls—1.00±0.44; for the 83 controls—0.76±0.62). Because there was no difference between the two groups, the two control groups were combined for further analyses. The PD patients were symptomatic for 3–5 years and had UPDRS-III scores from 3–50 (mean 17.09).

**Figure 1:**
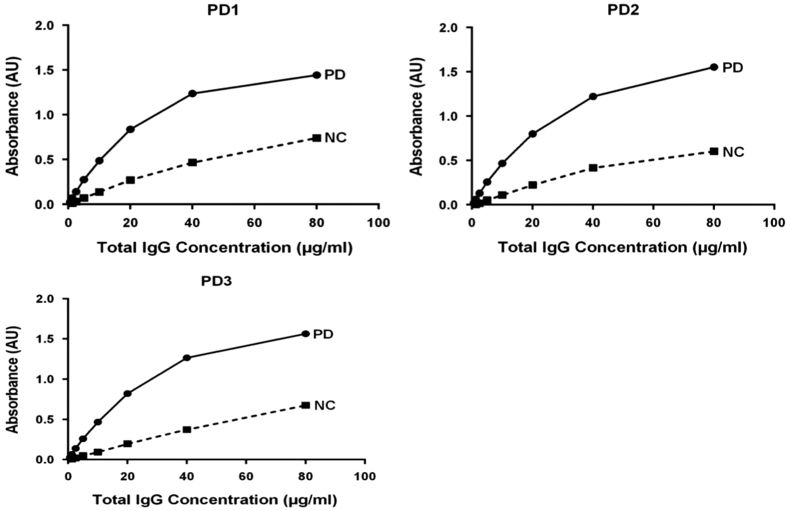
Peptoid binding to pooled samples from PD and NC subjects. Each of the three peptoids bound markedly higher levels of IgG in the PD pool versus normal control (NC) pool (*n*=20 per pool).

Three peptoid libraries were synthesized and screened for PD peptoids. Details of the libraries are illustrated in Table 2. Library 1 had a theoretical diversity=11,^7^ and was more hydrophobic than Library 2 (no Nlys residues). Library 3 had a theoretical diversity of 200,000 and Nlys residues were included. Peptoids from Library 2 were screened and three peptoids were identified using serum samples from a pool of PD subjects and a pool of normal control (NC) subjects (*n*=20 per pool and equal for male and female subjects). The three peptoids bound approximately twofold higher levels of IgG in the PD pool versus the NC pool (Figure 1). The PD2 peptoid was selected to test further for its ability to discriminate individual PD and NC samples.

**Figure 2:**
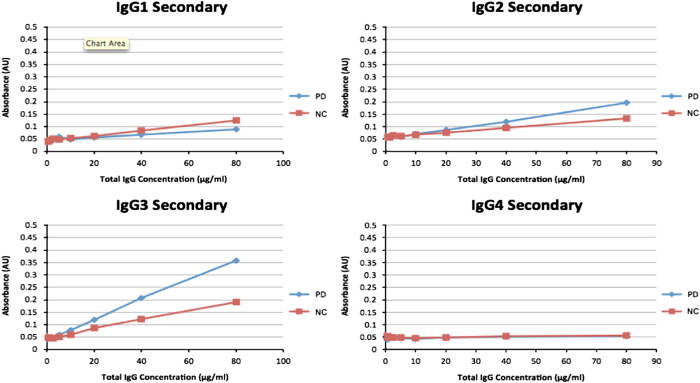
PD2 binding to IgG subtypes. Notice that among the four IgG (immunoglobulin G) subtypes the binding is selectively higher for PD (Parkinson's disease) versus normal control (NC) pool primarily for the IgG3 isotype.

We sought to determine which of the IgG subtypes the PD2 peptoid recognized. As shown in Figure 2, PD2 binds markedly higher levels of IgG3 from PD serum versus control serum. PD2 binding to the three other IgG subtypes is not marked different in PD versus control serum. Next we wanted to determine whether the levels of the IgG subtypes were different in PD subjects. Again, IgGs 1, 2 and 4 have similar levels in PD and control subjects (Table 3). However, levels of IgG3 are significantly higher in the PD subjects (*P*<0.001).

**Figure 3:**
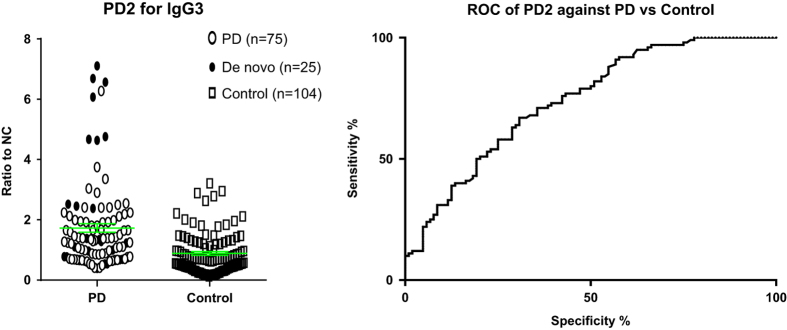
PD2 binding to individual PD and *De novo* patients. Left panel—binding levels are significantly higher in PD (*n*=75) versus vontrol (*n*=104) and *de novo* (*n*=25) versus control for PD2. Green bar=mean levels. Right panel—ROC curve for PD2 binding to PD versus Control subjects. AUC=0.74, *P*<0.001. PD, Parkinson's disease; AUC, area under the curve; ROC, receiver operating characteristic.

Using samples from individual PD and control subjects, levels of PD2 binding were found to be significantly higher in the PD patients (*P*<0.0001; Figure 3). We measured PD2 binding in 75 PD patients, 25 *de novo* PD patients, and 104 normal controls. Peptoid binding was also significantly higher in *de novo* PD subjects (*P*<0.0022; Table 4). The accuracy of the peptoid for identifying PD was 68%, but the accuracy for the peptoid identifying *de novo* PD was 84% (Table 5). For the *de novo* prediction the Sensitivity=0.40, Specificity=0.95, PPV=0.66 and NPV=0.86.

We also examined whether the PD2 peptoid was related to disease severity, as measured by the UPDRS. We found that the PD2 peptoid level for the PD subjects (*n*=75) was positively correlated with the UPDRS-III (*P*=0.014; *r*=0.283) and the UPDRS Total scores (*P*=0.034; *r=*0.245). In addition, we looked at the correlation between PD2 binding and UPDRS scores controlling for various PD medications taken by the patients using the Levodopa equivalent dose calculated according to Tomlinson *et al*.^15^ After controlling for differences in Levodopa equivalent dose among the PD patients, the PD2 peptoid was still highly correlated with the UPDRS scores (UPDRS-III: *P*=0.002; *r=*0.446; and UPDRS Total: *P*=0.0001; *r=*0.457).

## Discussion

Several peptoid libraries were used to search for an antibody biomarker for PD. These libraries contained ⩾200,000 different peptoids. We screened the peptoid libraries for IgGs that are elevated in PD serum using a magnetic screening approach. Five peptoids were found that bound higher levels of IgG in pooled serum from PD versus normal control subjects. We evaluated one of the PD peptoids, PD2, for IgG3 binding to individual samples from PD (*n*=75) and control (*n*=104) subjects. The PD2 peptoid was found to predict PD with an accuracy of 68%. This predictive accuracy is in line with other blood biomarkers for PD—e.g., uric acid^16^ and urate,^17^ apolipoprotein A1,^18^ and α-synuclein transcripts.^19^ For example, using three study cohorts α-synuclein was found to be lower in blood of *de novo* PD patients with an average AUC of 0.60. A panel of 50 autoantibodies was identified in the serum of *de novo* PD patients^20^ that was able to identify early-PD patients with an accuracy of 88%. Even using the top four autoantibodies in their panel they found similar accuracy, and these autoantibodies were able to distinguish PD from other neurodegenerative diseases, such as Alzheimer’s disease. It will be interesting to determine whether the PD2 peptoid is recognizing one of these four autoantibodies (microtubule affinity-regulating kinase 1, pseudouridylate synthase-like 1, interleukin-20, and chemokine (C–C motif) ligand 19), or possibly α-synuclein.^21^

It is interesting that IgG3 levels were significantly higher in PD patients versus normal controls. However, we found no correlation between PD2 binding and the specific patients’ serum IgG3 levels. This indicates that the PD2 peptoid recognizes an IgG3 antibody but it’s level is not representative of the total level of IgG3 in patient’s serum. Thus, the PD2 peptoid is not merely a marker for serum levels of IgG3.

The human IgG3 subclass is structurally different from IgG1, igG2, and IgG4 in that it has a much larger ‘hinge’ region between the CH1 and CH2 constant region domains (reviewed in Vidarsson, *et al.*^22^). The high proline content of the IgG3 hinge makes it much less flexible, and correlates with the binding of the complement C1q and Fc gamma receptors to the Fc region. This may increase the effector function of IgG3 relative to other IgG subclasses. IgG3 is also highly polymorphic between individuals. Many of these polymorphisms (allotypes) are in the hinge region and affect the serum half-life of the molecule, particularly through binding to the neonatal Fc receptror, FcRn.^23^ It is possible that PD2 binds to allotypic sequences in IgG3 that are more frequently expressed in PD individuals.

IgG3 has been postulated to have a role in neurodegenerative disorders. Elevated IgG3 was detected in 9 of 20 patients with Alzheimer’s disease compared to Down syndrome and age-matched controls,^24^ and 8 of these 9 patients had autoantibodies to brain tissue. IgG3 as well as IgG1 antibodies to neuronal antigens have been seen in several paraneoplastic neurological syndromes.^25,26^ Although IgG1 is the dominant subclass bound to dopamine neurons of the substantia nigra in post-mortem PD brain specimens, IgG3 was also detected.^7^

The PD2 peptoid was found to predict *de novo* PD with an accuracy of 84%. These 25 patients were early-stage patients defined with DAT-neuroimaging. This accuracy is close to the minimum recommended for a useful diagnostic test for neurodegenerative diseases.^27^ That the correlation between PD2 levels and UPDRS scores remained highly significant when controlled for amounts of dopaminergic medications suggests that this antibody is a marker of underlying disease pathology rather than an artifact of dopaminergic treatment. It is important to recognize that the data presented here represents a ‘discovery set’ and requires validation to be certain of its utility. Work is in progress to validate the PD2 peptoid for its ability to predict *de novo* PD and determine whether with a larger sample size both the Sensitivity and NPV scores are in a useful biomarker range.

### Conclusion

We have identified an antibody biomarker in the blood of PD patients using a combinatorial peptoid library approach. The peptoid approach to finding blood biomarkers has been used previously for systemic lupus erythematosus^28^ and autism spectrum disorder.^29^ The antibody is of the IgG3 subtype, is 84% accurate for the identification of *de novo* PD, and is positively correlated with disease severity (UPDRS score). Because the clinical identification of PD is often difficult, even in the early stage of the disease,^30^ a blood biomarker for the disease would be highly valuable. Additional studies are in progress to validate this peptoid PD biomarker.

## Materials and methods

### Human subjects

The study protocol and all subsequent amendments were approved by the Institutional Review Board at UT Southwestern (UTSW) Medical School. The study protocol was carried out in accordance with these approved guidelines.

The PD, *de novo* and some normal control subjects were participants in the NINDS PDBP at UT Southwestern Medical Center. Subjects were male or female age 50 years or older at time of PD diagnosis, Hoehn & Yahr (H&Y) Stage I–IV. Written informed consent was received from all subjects prior to enrollment. All PD patients met UK PD Society Brain Bank criteria,^31^ and were either *de novo* (previously untreated with dopaminergic medication) with a positive ioflupane iodine-123 SPECT scan (DATscan, Arlington Heights, IL, USA) consistent with degenerative parkinsonism, or were treated with dopaminergic drugs (levodopa or dopamine agonists) and known to be clinically responsive. In the case of the PD patients, all measurements were taken with the patients in the ON stage regarding PD medications. Exclusion criteria: (1) idiopathic PD, H&Y Stage V; (2) confirmed or suspected atypical parkinsonian syndromes due to drugs, metabolic disorders, encephalitis, or degenerative diseases; (3) presence of definite dementia (MoCA<17); and (4) any other medical or psychiatric condition or lab abnormality, which in the opinion of the investigator might preclude participation.

NC subjects were enrolled here at UT Southwestern Medical Center (*n*=21) and at other sites of the PDBP program (*n*=83), and were cognitively normal and free from neurodegenerative diseases based upon clinical evaluation, neuropsychological testing, and in some cases brain scans.

### Blood collection and storage

The blood collection methods are the same for all six of the PDBP fluid collection sites. Blood was collected into a 3.5-ml serum separation tube (Vacutainer System; Becton-Dickinson, Franklin Lakes, NJ, USA) using standard venipuncture technique. The blood was gently mixed in the serum separation tube by five inversions and then stored upright for clotting at room temperature for approximately 30 min. Blood was spun immediately after clotting in a swing bucket rotor for 20 min at 1,100–1,300*g* at room temperature. Serum was removed immediately after centrifugation and transferred into coded cryovials in 0.25-ml aliquots. Aliquots of serum were immediately placed upright in specimen storage box in a −20 °C freezer for up to 6 h. Samples were then transferred to a −80 °C freezer for long-term storage. Serum samples from normal control subjects collected at other PDBP sites were shipped to UTSW on dry ice. All blood samples were collected and stored according to protocols established by the Alzheimer’s Disease Neuroimaging Initiative (http://www.adni-info.org/Scientist/Pdfs/adni_protocol 9 19 08.pdf).

### Peptoid library synthesis

Three distinct one-bead one-compound combinatorial libraries of peptoids (oligo-N-substituted glycines) were synthesized onto 75 μm TentaGel beads using a split and pool method.^32^ Library 1 was configured as NH_2_-X_7_-Nmea-Nmea-Met-TentaGel, where X=Nall, Nasp, Ncha, Nffa, Nleu, Nmba, Nmpa, Nphe, Npip, or Nser, yielding a theoretical diversity of 10^7^ possible compounds. Monomer abbreviations: Met=methionine, Nall=allyamine, Nasp=glycine, Nbsa=4-(2-aminoethyl) benzenesulfonamide, Ncha=cyclohexylamine, Ndmpa=3,4-dimethoxyphenethylamine, Nffa=furfurylamine, Nippa=3-isopropoxypropylamine, Nleu=isobutylamine, Nlys=1,4-diaminobutane, Nmba=(R)-methylbenzylamine, Nmea=2-methoxyethylamine, Nmpa=3-methoxypropylamine, Nphe=benzylamine, Npip=piperonylamine, Npyr=N-(3′-aminopropyl)-2-pyrrolidinone, Nser=ethanolamine. Library 2 was configured as NH_2_-X_6_-Nmpa-Nlys-Met-TentaGel, where X=Nall, Nasp, Ncha, Nippa, Nleu, Nlys, Nmba, Npip, Npyr, Nser (theoretical diversity=10^6^ possible compounds). Library 3 was configured as NH_2_-X_5_-Nmea/Nlys-Ndmpa-Nmea-Met-TentaGel, where X=Nall, Nasp, Nbsa, Nippa, Nleu, Nlys, Nmba, Npip, Npyr, Nser (theoretical diversity=200,000 possible compounds). Methionine linkers were coupled in the usual way, whereas the peptoid residues were coupled using the submonomer method^33^ with microwave irradiation to accelerate reactions.^34^ Proper library syntheses were confirmed by CNBr cleavage of compounds from samples of isolated beads and subsequent analysis by tandem mass spectrometry.

### On-bead magnetic screening

**Figure 4:**
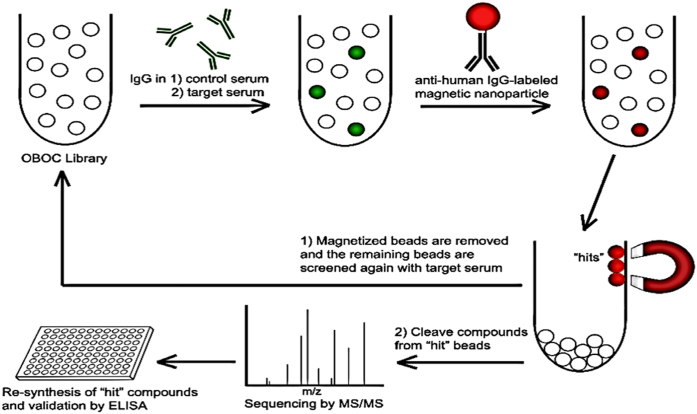
On-bead magnetic screening. A one-bead one-compound (OBOC) library of thousands of unique peptoid compounds bound to TentaGel beads is incubated with control serum, here serum pooled from normal control subjects. The library is then incubated with anti-human IgG-labeled magnetic nanoparticles so that beads having bound IgG from the serum can be sorted out using a strong magnet. The library is initially depleted of beads that bind IgG from the control serum, and then incubated with target serum, here serum pooled from PD subjects. After incubation with the magnetic nanoparticles again, the newly magnetized beads, called ‘hits’, are isolated. Peptoid compounds are cleaved from each of the ‘hit’ beads and their sequences are assessed by MS/MS. These ‘hit’ compounds are then resynthesized and validated on ELISA plates for their ability to detect target IgG. (Reprinted by permission from Macmillan Publishers: from Zaman *et al.*^29^). ELISA, enzyme-linked immunosorben assay.

A modification of the magnetic capture method for screening one-bead libraries was used.^35^ Approximately 375,000 beads from the library were soaked in PBST (PBS-0.1% Tween 20, pH 7.4) and then blocked with blocking buffer (1:1 mixture of 1% bovine serum albumin (BSA) in PBST and SuperBlock Blocking Buffer (Thermo Scientific, Rockford, IL, USA) for 1 h. at room temperature (RT). Peptoid screening always used serum samples from pooled PD and NC subjects. Serum aliquots from 20 control subjects were pooled and then diluted up to 1 ml in blocking buffer to obtain a final IgG concentration of 40 μg/ml. IgG levels of serum pools were measured using Human IgG ELISA Quantitation Set (Bethyl Laboratories, Montgomery, TX, USA). The library beads were then incubated with the diluted serum in a cryovial overnight at 4 °C. The beads were washed with PBST eight times and resuspended in blocking buffer. A 10 mg/ml anti-human IgG-conjugated Dynabead solution, 50 μl, was prepared by coupling 10 μg of biotinylated Goat F(ab)_2_ anti-human IgG (Southern Biotech, Birmingham, AL, USA) with 0.5 mg of Dynabead M-280 Streptavidin (Invitrogen, Grand Island, NY, USA). The library beads were mixed with the Dynabead solution for 2 h. at RT with gentle agitation. Library beads with high levels of bound Dynabeads were then separated by placing the tube in a strong magnetic field. These ‘magnetized’ beads were removed from the library. The remaining beads were again washed and the magnetic capture was repeated two more times, completing the depletion. The depleted library was then incubated with pooled serum aliquots from 20 PD subjects as described above. ‘Hit’ beads were obtained by performing the magnetic capture and collecting the magnetized beads. ‘Hit’ peptoid compounds were then identified by CNBr cleavage of compounds from ‘hit’ beads and sequencing by MALDI TOF/TOF (Figure 4).

For validation and subsequent analyses, ‘hit’ peptoid compounds were resynthesized on Polystyrene AM RAM resin (Rapp Polymer, Tübingen, Germany) with the methionine linker, as in the library, replaced by a cysteine linker so that the compounds could be immobilized using sulfhydryl-reactive chemistry. Peptoid compounds were cleaved off the resin by incubating in a 95% trifluoroacetic acid, 2.5% triethylsilane, 2.5% water solution for 2 h. at RT. Peptoid compounds were subsequently purified using high-performance liquid chromatography and verified by MS analysis.

### Peptoid ELISA

The PD2 peptoid was immobilized onto maleimide-activated 96-well plates (Pierce Biotechnology, Rockford, IL, USA) by dissolving them to 0.03–0.05 mmol/l in a 0.1 mol/l sodium phosphate, 0.15 mol/l sodium chloride, 10 mM EDTA solution adjusted to pH 7.2 and incubating with shaking for 3 h. at RT. Plates were washed with PBST and blocked with a 5% goat serum (Thermo Scientific, Rockford, IL) in PBST solution for 1 h. at RT. Plates were washed again and incubated with target (serum) samples diluted in blocking buffer (1:1 PBST-1%BSA and SuperBlock) for 2 h. at RT. After washing, plates were incubated with Goat anti-Human IgG-Fc-HRP conjugate (Bethyl Laboratories, Montgomery, TX, USA) diluted 1:30,000 in PBST-1%BSA *or* mouse anti-human IgG3 (hinge)-HRP conjugate (SouthernBiotech, Birmingham, AL, USA) diluted 1:1,000 in PBST-1%BSA for 30 min at RT. After another wash, plates were incubated in TMB substrate for 16 min at RT and stopped with 2 mol/l H_2_SO_4_. Plates were read at 450 nm. All samples were run in duplicate, and every assay contained PD and NC serum pool samples to serve as internal controls. Results for individual samples were assessed as ratios to the NC serum pool so as to control for plate-to-plate variation.

For control experiments, total IgG levels for individual serum samples were quantified using human IgG ELISA Quantitation Set (Bethyl Laboratories, Montgomery, TX, USA), and IgG1–4 levels were quantified using IgG Subclass Human ELISA Kit (Invitrogen, Grand Island, NY, USA).

### Statistics

Statistical analyses were performed using GraphPad Prism 6 (San Diego, CA, USA) and IBM SPSS Statistics V19 (New York, NY, USA). The mean values of untransformed ELISA (enzyme-linked immunosorbent assay) data for individual samples were compared by Kruskal–Wallis *H*-test, and Mann–Whitney *U*-tests. Peptoid binding was regressed on the United Parkinson’s Disease Rating Scale (UPDRS)-II, UPDRS-III, and UPDRS Total subdomain scores to examine whether the PD2 peptoid binding was related to disease severity as measured by the UPDRS. Prior to fitting regression models, peptoid binding was square-root-transformed to reduce the positive skew; the transformed distribution was approximately normally distributed and met guidelines for covariance matrix based models.^36^ ROC and cutscore methods were used to determine the accuracy of the PD2 peptoid for predicting PD versus NC. Medians and range were used to describe the two groups of PD and NC subjects and the Mann–Whitney *U*-test was used to compare groups on PD2 peptoid binding levels. To explore the use of a cutscore for group prediction, ROC analysis was used. Several criteria were used to define the optimal cutscore for these data; (1) the maximum perpendicular distance from (and above) the 45 degree line of equality,^37^ (2) highest accuracy (correct predictions), and (3) best sensitivity/specificity combination. Chi-square test of independence was used to examine the predictions using the new cutscore.

## Figures and Tables

**Table 1:** Demographics of patient population

	*PD*	*Control*	*De novo*
Age (years±s.d.)	69±5	69±5	62±10
Gender (% male)	51	51	48
*N*	75	104	25

Abbreviation: PD, Parkinson's disease.

**Table 2:** Peptoid libraries screened

*Serum pools*	*Library*	*Peptoids assessed*
PD, *n*=6 males	1	10 Peptoids
NC, *n*=6 males		Named PD1E—PD10E
PD, *n*=20 (10 males, 10 females)	2	3 Peptoids
NC, *n*=20 (10 males, 10 females)		Named PD1—PD3
PD, *n*=40 (20 males, 20 females)	3	4 Peptoids
NC, *n*=22 (11 males, 11 females)		Named PD4—PD7

Abbreviations: NC, normal control; PD, Parkinson’s disease.

**Table 3:** IgG subtype levels in PD

*Human IgG*	*PD (*n*=69)*	*Control (*n*=53)*
IgG1	8.265±2.862	8.192±3.314
IgG2	2.941±1.315	3.419±1.351
IgG3	1.029±0.696	0.701±0.391**
IgG4	0.365±0.325	0.441±0.402

Abbreviation: IgG, immunoglobulin G; PD, Parkinson's disease.

Mean±s.d. **P=0.0023 (Mann–Whitney test).

**Table 4:** PD2 binding is higher in PD and *De Novo* patients versus Control subjects

*Group*	*PD2 Levels*					*Groups*	*PD2 Levels*
	N	*Mean*	*s.d.*	*Min*	*Max*		*Mann–Whitney* P *value*
Control	104	0.89	0.68	0.12	3.21		
PD	75	1.49	0.92	0.41	6.28	PD versus control	<0.0001
PD+*de novo*	100	1.73	1.43	0.38	7.11	PD+*De novo* versus control	<0.0001
*De novo*	25	2.45	2.26	0.38	7.11	*De novo* versus control	0.0022

Abbreviation: PD, Parkinson's disease.

**Table 5:** PD2 predicts PD with high accuracy for *de novo* patients

*PD2 levels*								
*Group*	*Area under the curve*					*Best cutscore*	P *value*	*Accuracy*
	*Area*	*s.e.*	P *value*	*Asymptotic 95% confidence interval*				
				*Lower bound*	*Upper bound*			
PD versus control	0.738	0.036	<0.0001	0.667	0.810	0.9755	<0.0001	0.6872
PD+*de novo* versus control	0.74	0.034	<0.0001	0.673	0.806	0.9755	<0.0001	0.6814
*De novo* versus control	0.744	0.054	0.0002	0.639	0.849	2.294	<0.0001	0.845

Abbreviation: PD, Parkinson's disease.
